# Revisiting In‐Gas Transformations of Quinate Conjugates Through the LC‐qTOF‐MS and Molecular Networking Topology

**DOI:** 10.1002/rcm.10068

**Published:** 2025-05-08

**Authors:** Nakisani Babra Moyo, Ntakadzeni Edwin Madala

**Affiliations:** ^1^ Department of Food Science and Technology, Faculty of Science, Engineering and Agriculture University of Venda Thohoyandou South Africa; ^2^ Department of Biochemistry and Microbiology, Faculty of School of Science, Engineering and Agriculture University of Venda Thohoyandou South Africa

**Keywords:** chlorogenic acids, in silico predictor, mass spectrometry, molecular networking topology, quinates

## Abstract

**Rationale:**

The emergence of computational metabolomics tools such as molecular networking and machine learning–based platforms like SIRIUS has significantly advanced MS‐based metabolomics studies. These tools enable rapid metabolite identification by deciphering complex fragmentation patterns and chemical transformations occurring during mass spectrometry analysis.

**Methods:**

In this study, methanolic extracts of *Viscum combreticola*, a plant recently shown to contain a rich composition of cinnamic acid–quinates conjugates, were analyzed using the LC‐qTOF‐MS in combination with a molecular networking approach to explore the chemical complexity of quinate conjugates.

**Results:**

Findings of this study through molecular networking topology revealed that quinic acid undergoes a series of in‐gas chemical transformations, including dehydration (–H_2_O) and decarboxylation (–CO_2_). These transformations yield unique product ions, some of which are associated with other organic acids, such as isocitric acid. By employing the MS^2^ search option on the GNPS2 platform, molecules exhibiting these product ions were readily identified in this study. Therefore, highlighting the potential of this function in GNPS2 for tracing unique fragmentation patterns synonymous with certain molecules that can be used to confirm their identity visually.

**Conclusion:**

The MS^2^ search function can aid in the discovery of new compounds containing the diagnostic ions of interest that could otherwise be easily missed with manual annotation. This study presents a potential validation approach of looking at multiple product ions to confirm the identity of a molecule, particularly in the presence of other compounds with similar fragmentation pathways or shared fragment ions.

## 1 Introduction

Hundreds and thousands of secondary metabolites can be detected through liquid chromatography tandem mass spectrometry (LC–MS/MS) from various plant samples. However, the complexity and wide diversity of phytochemicals continue to present significant challenges for the identification and classification of these compounds in plant metabolomics workflows [1, 2]. Of the available acquisition modes for metabolomics using the LC–MS/MS, the data‐dependent acquisition (DDA) mode has been reported to provide higher quality spectra that can significantly improve compound annotation through in silico tools because the resulting full‐scan (MS) and MS/MS spectra are synchronized [3, 4]. To match advancements in analytical instrumentation, various metabolomics computational tools including in silico fragmentation predictors [5, 6, 7] and molecular networking [8, 9] have emerged in the past years, marking an enormous milestone in the field. These tools mine MS/MS fragmentation patterns from complex mixtures and assign putative annotations to compounds, which can be propagated to structurally related molecules [7, 10].

Computational metabolomics tools also offer a significant advantage of providing infographic displays of MS/MS fingerprints, facilitating the identification of features of interest across the entire plant metabolome. For instance, the MS^2^ search function available on GNPS2 is an incredibly powerful function that can search and display specific MS/MS spectra or fragmentation patterns across the whole dataset. Similar to MS2LDA [11], the MS^2^ search function identifies co‐occurring structural motifs, thereby allowing for the detection of structural similarities even among chemically diverse molecules. Therefore, this function can be exploited to accelerate the discovery of new bioactive compounds for various applications. On the other hand, SIRIUS simulates the sequential fragmentation of molecular ions which resembles the “in time” fragmentation observed in ion trap instruments even when using other types of mass spectrometers such as the qTOF where fragmentation occurs “in space.” This allows a comprehensive analysis of fragmentation pathways of compounds, hence giving detailed structural insights into the molecules of interest. These computational metabolomics advancements are particularly valuable when analyzing complex classes of plant metabolites like chlorogenic acids (CGAs) which exhibit structural diversity and closely related fragmentation patterns.

CGAs are among the common polyphenols found in plants and are particularly abundant in coffee, where a diverse range of these compounds have been identified [12, 13]. A recent study by Moyo et al. [14] also identified several of these structurally diverse compounds from an African mistletoe, *Viscum combreticola*. Despite the structural diversity of CGAs, the hierarchical fragmentation scheme keys devised by Clifford et al. [12] using ion trap mass spectrometry (IT‐MS) have made differentiating between isomers of the same compound easy based on their fragmentation patterns. The numerous therapeutic properties of CGAs, such as antioxidant [15], anti‐inflammatory [16], and potential antidiabetic effects [17], make their accurate identification crucial for understanding their biological roles and potential applications. Therefore, establishing additional compound identification approaches that enable the precise differentiation of structurally similar metabolites is essential for advancing research into their pharmacological and nutraceutical potential, particularly given the structural complexity of CGAs and the occurrence of common fragment ions across different compound classes that can be found in a one sample.

*V. combreticola* has been found to exhibit an overwhelming presence of the product ion at *m/z* 137 where it was reported to be associated with different compound classes. In a study by Moyo et al. [8], this fragment ion was attributed to the retro‐Diels–Alder reaction of flavan glycosides. In another study by Moyo et al. [14], *m/z* 137 was identified as a characteristic fragment ion of hydroxybenzoyl containing CGAs, corresponding to the deprotonated hydroxybenzoic acid ion ([M–H]^−^ = 137). In the present study, this ion was further observed as a product ion of QA derivatives, necessitating the need to differentiate its origin in complex mixtures. Therefore, in this study, liquid chromatography quadrupole‐time‐of‐flight mass spectrometry (LC‐qTOF‐MS) coupled to molecular networking topology and SIRIUS were used to show for the first time a unique fragmentation pattern of quinic acid (QA) that occurs when QA is acylated at position carbon 4 (C4) using CGAs from *V. combreticola* as model compounds. 3,4‐Digallolyquinic acid identified in *Tapinanthus quequensis* was used to further strengthen these findings. The fragmentation pattern of QA was also compared to that of its isobar, isocitric acid.

## 2 Materials and Methods

### 2.1 Sample Preparation and Analysis on the UHPLC‐qTOF‐MS

Samples of *V. combreticola* (whole plant) were collected from Shakadza, Limpopo, South Africa, and prepared according to a method described by Moyo et al. [14]. Briefly, approximately 2 g of dry and ground samples were extracted in 20 mL of 80% methanol (1:10 *m/v*) using a digital tube rotator (Dlab Scientific, Beijing, China) at 70 rpm overnight. The resulting crude extracts were centrifuged at 2739 × *g* using a benchtop fixed‐angle centrifuge (Thermo Fisher, Johannesburg, South Africa), and the supernatants were filtered through 0.22‐μm nylon filters into amber HPLC vials for analysis. *T. quequensis* and *Momordica balsamina* were also prepared using the same method for supporting the QA fragmentation pattern discussed in this study.

LC‐qTOF‐MS analysis was performed using a Shimadzu 9030 system according to a method by Moyo et al. [14]. Chromatographic separation was achieved by injecting 5 μL of the filtered extracts onto a Shim‐pack Scepter C_18_ column (100 × 2.1 mm, 1.9 μm, Shimadzu Corporation, Kyoto, Japan) maintained at 55°C. A binary gradient mobile phase was used at a flow rate of 0.3 mL min^−1^, with mobile phase A consisting of 0.1% (v/v) formic acid in ultrapure water and mobile phase B comprising 0.1% (v/v) formic acid in methanol. The gradient program was as follows: 10% mobile phase B for 0–3 min, held steady until 40 min, increased to 60% from 40 to 43 min, further increased to 90% between 45 and 49 min, and returned to 10% mobile phase B at 50 min, maintained until 53 min.

Mass spectral analysis was conducted using a high‐resolution qTOF mass spectrometer equipped with an electrospray ionization interface (ESI) operating in negative ionization mode. The instrument parameters included an interface voltage of 4.0 kV, an interface temperature of 300°C, a nebulization and dry gas flow rate of 3 L min^−1^, a heat block temperature of 400°C, a DL temperature of 280°C, a detector voltage of 1.8 kV, and a flight tube temperature of 42°C. Sodium iodide was used as the calibration solution to ensure accurate mass determination. Ions within an *m/z* range of 100 to 1000 Da and intensities exceeding 5000 were selected for analysis. MS^1^ and MS^2^ spectra were simultaneously acquired using DDA mode with argon as the collision gas. A collision energy of 30 eV with a spread of ±5 was applied to generate MS/MS fragmentation data for compound identification and structural elucidation.

### 2.2 Molecular Networking and In Silico Fragmentation Pattern Prediction

Classical molecular networking was done on the GNPS2 (https://gnps2.org) platform from raw data in mzML format that was uploaded on the online workflow. Mass tolerances for precursor and MS^2^ fragment ion were set to 0.02 Da. A cosine score threshold of 0.6 was used to evaluate spectral similarity with a minimum of four matched fragments for inclusion in the network. The resulting molecular network was visualized with Cytoscape software [18]. SIRIUS 5.5.7 was used to predict fragmentation patterns of the compounds of interest in this study [7]. Data in mzML format was imported to SIRIUS 5.5.7. For molecular formula identification, negative ionization was selected for possible ionization, the MS/MS isotope scorer was set on score, and the elements allowed in the molecular formula were C, H, and O. Structural database search was done on the databases including GNPS, HMDB, Knapsack, Coconut, CHEBI, and Natural Products. Canopus was used for compound class prediction.

## 3 Results and Discussion

**FIGURE 1:**
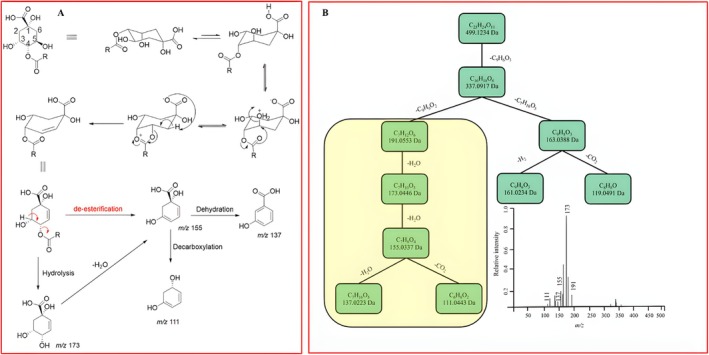
Proposed unique fragmentation pathway mechanism of quinic acid in negative ionization (A) and predicted fragmentation pattern of 3‐coumaroyl‐4‐caffeoylquinic acid on Sirius (B).

Exploring novel compounds from plants and other natural sources forms the cornerstone of discovering new therapeutic agents. Moreover, correctly identifying these compounds is critical for understanding their potential therapeutic properties. A unique fragmentation pattern of QA in CGAs from *V. combreticola* using the LC‐qTOF‐MS in negative ionization coupled to molecular network topology was unveiled in this study. Notably, QA‐containing compounds showed a unique fragmentation pattern resulting from QA when acylated at position C4 of the QA molecule. To the best of our knowledge, this is the first study that showed that QA undergoes triple dehydration when QA is conjugated at position C4 (Figure 1A), and these can be used as diagnostic fragment ions to characterize isomeric QA‐containing molecules. QA‐containing molecules acylated at position C4 of QA are normally identified by the presence of an intense fragment ion at *m/z* 173 that results from the dehydration of QA which has a deprotonated molecular ion [M–H]^−^ at *m/z* 191 [12]. Zhang et al. [19] showed that QA produces a fragment ion at *m/z* 137; however, the proposed fragmentation was not detailed. The proposed detailed fragmentation mechanism of QA as observed from qTOF‐DDA‐MS data is shown in Figure 1A, where after an initial dehydration of QA (*m/z* 191), a product ion at *m/z* 173 is formed which further dehydrates to a product ion at *m/z* 155. This product ion at *m/z* 155 further loses another water molecule and results in the formation of hydroxybenzoic acid (*m/z* 137). On the other hand, when a product ion of QA at *m/z* 155 decarboxylates, a fragment at *m/z* 111 is formed. This fragmentation pattern was further confirmed using SIRIUS, as shown in Figure 1B in 3‐coumaroyl‐4‐caffeoylquinic acid at *m/z* 499.123. Interestingly, using a qTOF‐MS from Waters, the same fragmentation pattern except the presence of the product ion at *m/z* 137 was also noted for isocitric acid/citric acid derivatives; however, the products ions (*m/z* 155, 137, and 111) were not present on the QA‐containing molecules [20]. Therefore, the presence of the product ion at *m/z* 137 associated with QA derivatives is an interesting phenomenon that can be used to further strengthen the identification of QA‐containing molecules, building on the already reliable hierarchical fragmentation keys [12]. It is noteworthy that the initial method of identification of these molecules was previously accomplished through ion trap–based instrumentation but as technology advances; other forms of mass analyzers such as qTOF‐MS have also been shown to produce similar fragmentation patterns [21, 22]. Most importantly, the cascade of fragmentation events displayed in Figure 1B is also consistent with previous findings, even though the presence of the *m/z* 137 product ion was only noted in one of the four synthesized stereoisomers of QA [23].

**FIGURE 2:**
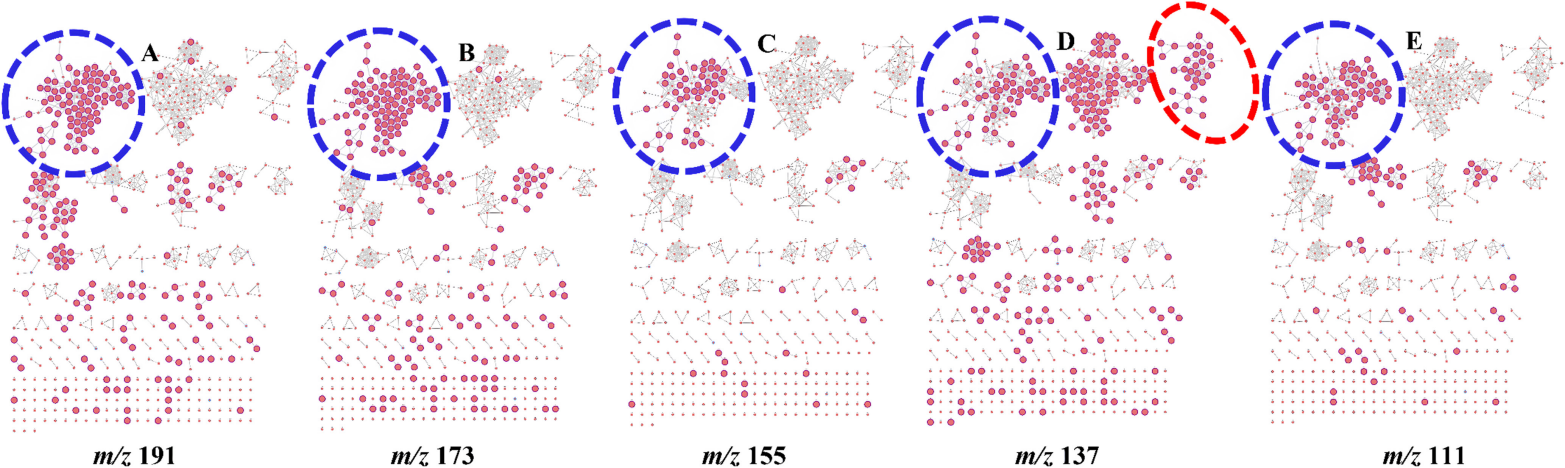
Infographic presentation of the full molecular network showing the fragmentation pattern of quinic acid (*m/z* 191) (A) containing molecules using the MS^2^ *m/z* highlight function on GNPS2 showing MS^2^ ions resulting from triple dehydration of QA resulting in product ions at *m/z* 173 (B), 155 (C), and 137 (D), respectively. A product ion resulting from double dehydration followed by decarboxylation (*m/z* 111) is shown on (E). The CGAs molecular family is circled in blue dashed lines whereas the one circled in red in (D) is for flavan glycosides.

**FIGURE 3:**
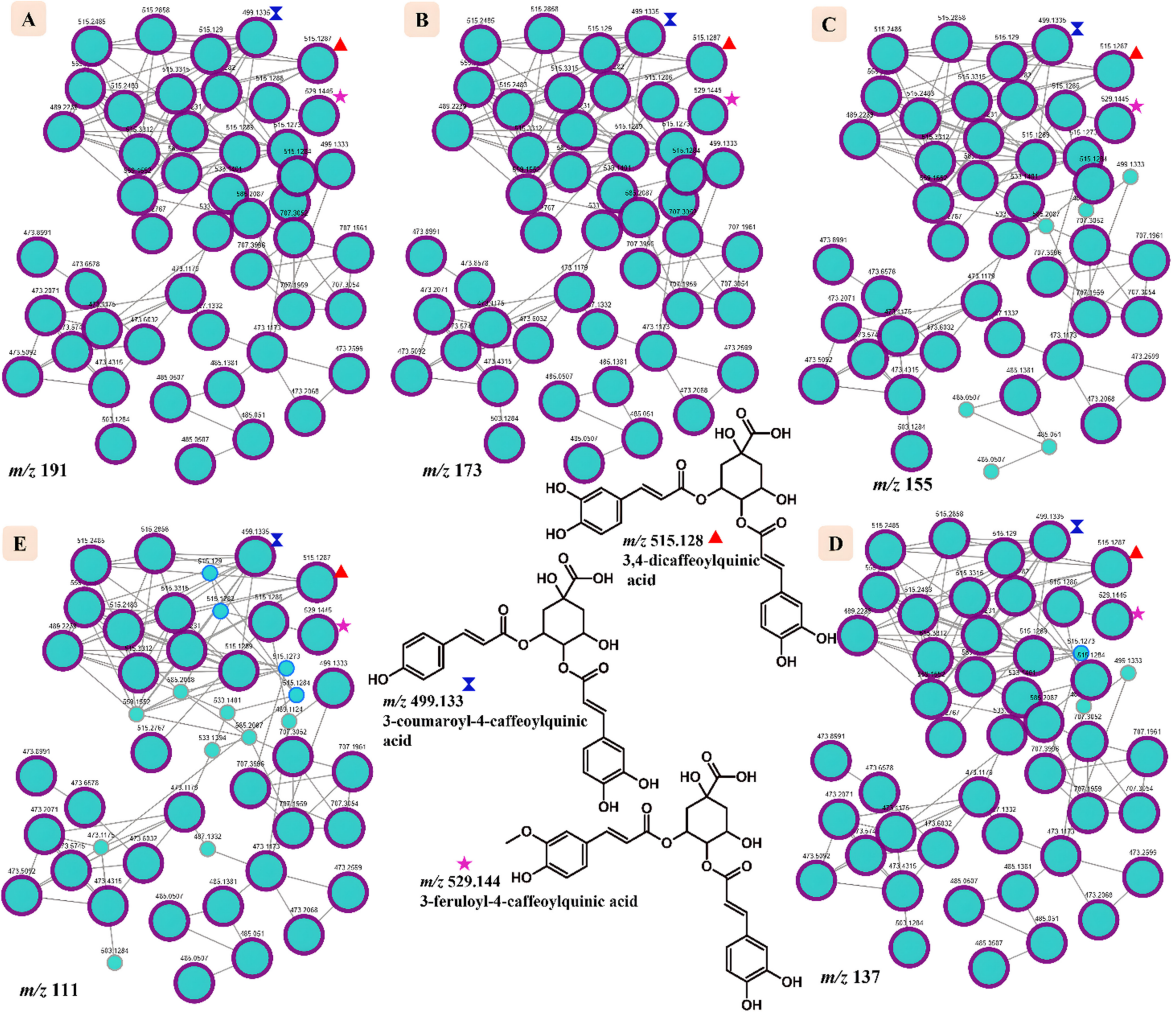
Infographic presentation of CGAs cluster showing the fragmentation pattern of quinic acid (*m/z* 191) (A) in CGAs where QA is acylated at position C4 using molecular networking showing MS^2^ ions resulting from triple dehydration of QA resulting in product ions at *m/z* 173 (B), 155 (C), and 137 (D), respectively. A product ion resulting from double dehydration followed by decarboxylation at *m/z* 111 (E). The shapes in different colors (blue, red, and pink) next to nodes representing *m/z* 499.133, 515.128, and 529.144 are used to map the nodes to their compound structures.

Employing molecular networking topology in Figures 2 and 3, it was also demonstrated that unique fragmentation pathways of compounds can be tracked infographically to ascertain their correct identity in complex samples containing molecules that fragment in a similar pattern. In Figure 2, characteristic fragment ions of QA resulting from triple dehydration (*m/z* 173, 155, and 137) and decarboxylation (*m/z* 111) of this moiety in the CGA cluster proposed in Figure 1A are shown through molecular networking. Through the MS^2^ highlight function, nodes that exhibit this fragmentation pattern are highlighted showing their consistent presence from *m/z* 191 to 111. Figure 3 shows that indeed CGAs acylated at position C4 of QA undergo this unique fragmentation cascade, and this was demonstrated using compounds at *m/z* 499.123, 515.128, and 529.144. This was also supported by the same fragmentation of QA in 3,4‐digalloylquinic acid identified in *T. quequensis* as shown in Figure S1, hence showing that this fragmentation pattern is consistent regardless of the moiety attached to QA. The identity of CGAs with deprotonated molecular ions [M–H]^−^ at *m/z* 515.128 (3,4‐dicaffeoylquinic acid) and 529.144 (3‐feruloyl‐4‐caffeoylquinic acid) in Figure 3 have been extensively characterized in a study done by Moyo et al. [14] from MS/MS data; therefore, their fragmentation patterns will not be discussed further in this study.

Given that structural similarities and overlapping fragmentation patterns stemming from shared biosynthetic pathways among certain compounds can result in misidentifications, the potential of computational metabolomics tools as valuable tools for distinguishing closely related compounds in complex mixtures including isobaric molecules was also highlighted in this study. The fragmentation patterns of 4‐caffeoylquinic acid (*m/z* 353.085) and 2‐caffeoylisocitrate (*m/z* 353.054) were also compared using SIRIUS to further strengthen the uniqueness of QA's fragmentation pattern. As shown in Figure S2, isocitric acid and QA are not only isobaric but they also share common fragmentation pathways with the exception of the product ion at *m/z* 137, observed only in QA. This, therefore, highlights that the inspection of unique fragmentation patterns using computational tools can be infographically explored to track diagnostic peaks of isobaric compounds. This approach not only enhances the dereplication of compounds but can also be a powerful infographic tool for visualizing unique fragmentation pathways of compounds. These visualizations can be used for distinguishing closely related or isobaric compounds and may facilitate the discovery of new ones. In particular, molecular networking through the MS^2^ search function that can search for diagnostic peaks of interest throughout the whole data set that can otherwise be missed through manual annotation. Although molecular networking does not currently show chemical transformations such as dehydration and decarboxylation and other in‐gas reactions occurring within the MS, integrating complementary tools such as SIRIUS can reveal these processes visually (Figure 1B). Therefore, this underscores the need for advanced molecular networking algorithms capable of visualizing such transformations, which can also be extended to study other biological modifications, including phosphorylation, glucuronidation, methylation, and hydroxylation.

The MS^2^ search function, as demonstrated in this study, should however be used with caution given that some natural products are isobaric, which can lead to misidentification, as shown by the case of QA and isocitric acid (Figure S2) [20]. Also, in our recent findings, we demonstrated that a motif at *m/z* 137 originates from a retro‐Diels–Alder reaction of viscutin flavonoids found in *V. combreticola* [8]. However, in the current study, an isobaric peak at *m/z* 137 has also been identified as a diagnostic ion for QA derivatives in the same plant. This occurrence of the same fragment/product ions in different molecules presents a potential source of confusion in distinguishing between these structurally distinct compounds. Therefore, this study presents a potential validation approach by considering multiple product ions in the identification process. These additional product ions, referred to as “qualifier ions,” should be consistently present to confirm the identity of a given molecule. Specifically, in our present study through molecular networking (Figure 2 and 3), not all nodes exhibiting the presence of the ion at *m/z* 191 (presumed to be QA) also displayed the expected qualifier ions at *m/z* 173, 155, 137, and 111 (Figure 2). Therefore, only those molecular features that consistently exhibit the full set of qualifier ions can be reliably classified as QA‐containing compounds. Additionally, in silico tools for predicting fragmentation patterns can serve as complementary tools to molecular networking that can be used to further support and validate experimental data. Therefore, molecular networking and in silico predictors represent transformative tools in natural product research, improving our ability to identify and differentiate complex metabolites and accelerating the discovery of potential novel therapeutic agents.

## 4 Conclusion

In this study, the integration of high‐resolution MS with computational metabolomics tools proved to be a robust approach for elucidating a distinctive fragmentation pathway of QA. While the specific fragmentation patterns of QA may vary depending on instrumentation and associated parameters, these findings highlight the effectiveness of combining high‐resolution MS with molecular networking topology in confirming the identity of quinates or any other compounds in complex metabolite profiles, enabling the precise characterization of structurally related natural products. By using the MS fragmentation visualization such as SIRIUS, new diagnostic ions synonymous with certain isomeric forms of pharmacologically relevant metabolites could be rationalized. The analytical and computational metabolomics pipeline presented in this study is not limited to QA‐containing compounds, it can be adopted to investigate fragmentation patterns of other compound classes across a variety of matrices. Also, the results of the current study further extend on the already established ion trap MS‐based fragmentation keys of QA‐containing molecules and provide an orthogonal view which can be used to visualize the stepwise fragmentations of molecules using “in space”–based fragmentation instruments such as qTOF‐MS.

## Author Contributions

**Nakisani Babra Moyo:** writing – original draft, data curation, investigation, formal analysis. **Ntakadzeni Edwin Madala:** conceptualization, writing – review and editing, methodology.

## Conflicts of Interest

The authors declare no conflicts of interest.

### Peer Review

The peer review history for this article is available at https://www.webofscience.com/api/gateway/wos/peer‐review/10.1002/rcm.10068.

## Supporting information


**Figure S1.** Fragmentation pattern presentation of quinic acid (*m/z* 191) in 3,4‐digalloylquinic acid using molecular networking. The blue diamond next to the node representing *m/z* 495.077 is used to map the node to its compound structure.
**Figure S2.** Comparison of the fragmentation patterns of quinic acid in 4‐caffeoylquinic acid (A) and isocitric acid in 2‐caffeoylisocitrate (B).

## Data Availability

The data that support the findings of this study are available from the corresponding author upon reasonable request.
