# Supplementary material for: Revisiting In‐Gas Transformations of Quinate Conjugates Through the LC‐qTOF‐MS and Molecular Networking Topology
Source: Rapid Commun Mass Spectrom. 2025 May 8;39(16):e10068. doi: 10.1002/rcm.10068 (PMC12062773; doi:10.1002/rcm.10068)
Supplement: Supplementary file 1 — Figure S1. Fragmentation pattern presentation of quinic acid (m/z 191) in 3,4‐digalloylquinic acid using molecular networking. The blue diamond next to the node representing m/z 495.077 is used to map the node to its compound structure. Figure S2. Comparison of the fragmentation patterns of quinic acid in 4‐caffeoylquinic acid (A) and isocitric acid in 2‐caffeoylisocitrate (B). [file RCM-39-e10068-s001.docx]

**Supplementary information**

**GNPS job links**

*Viscum combreticola* job link: <https://gnps2.org/status?task=dfefe6309f124df8a18e0c95708c48b8>

*Tapinanthus quequensis* job link: <https://gnps2.org/status?task=a374e144bc444a3aa3ebd73bcc95c0c5>

**Supplementary figure**

**
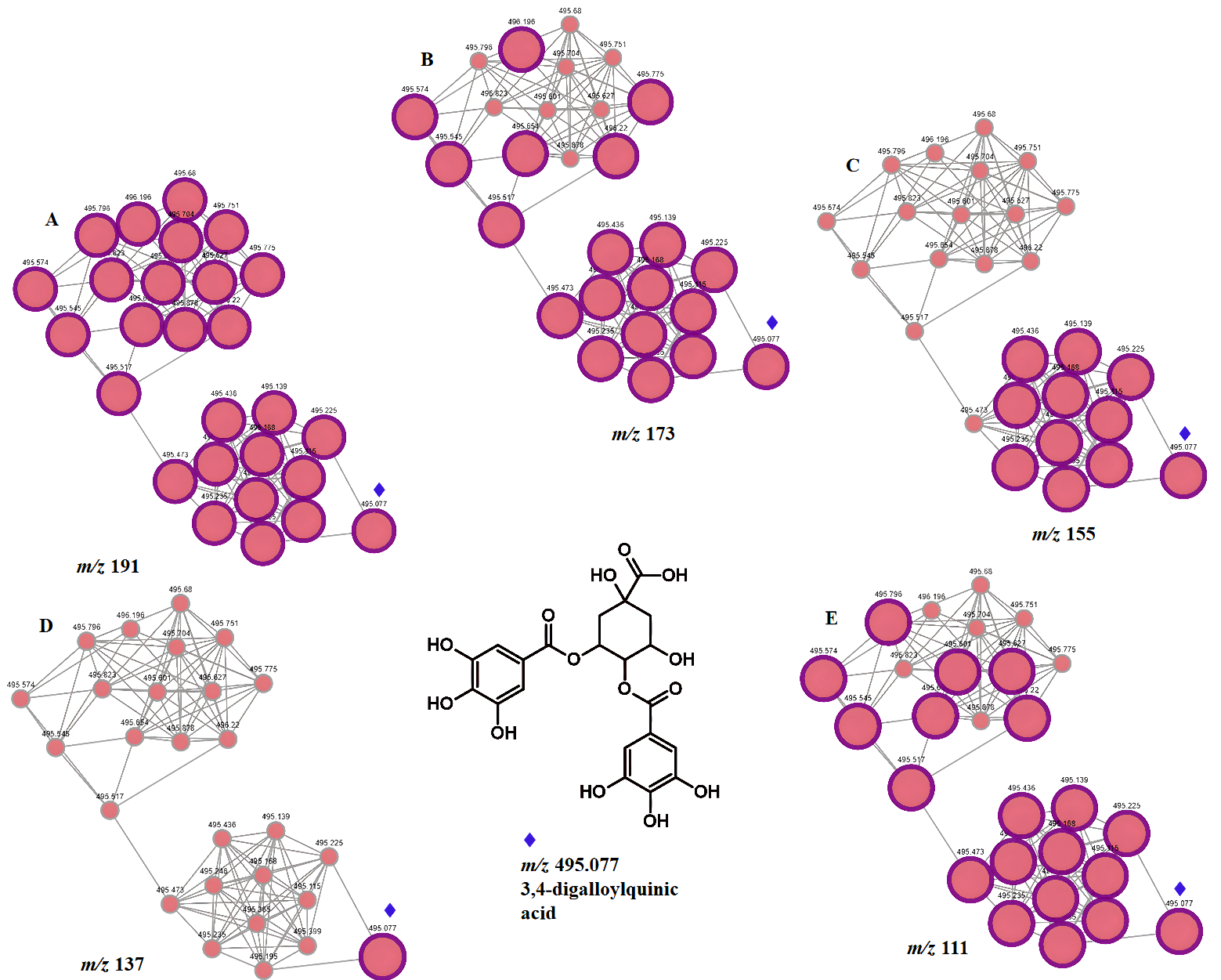
**

**Figure S1 F**ragmentation pattern presentation of quinic acid (*m/*z 191) in 3,4-digalloylquinic acid using molecular networking. The blue diamond next to the node representing *m/z* 495.077 is used to map the node to its compound structure.

**
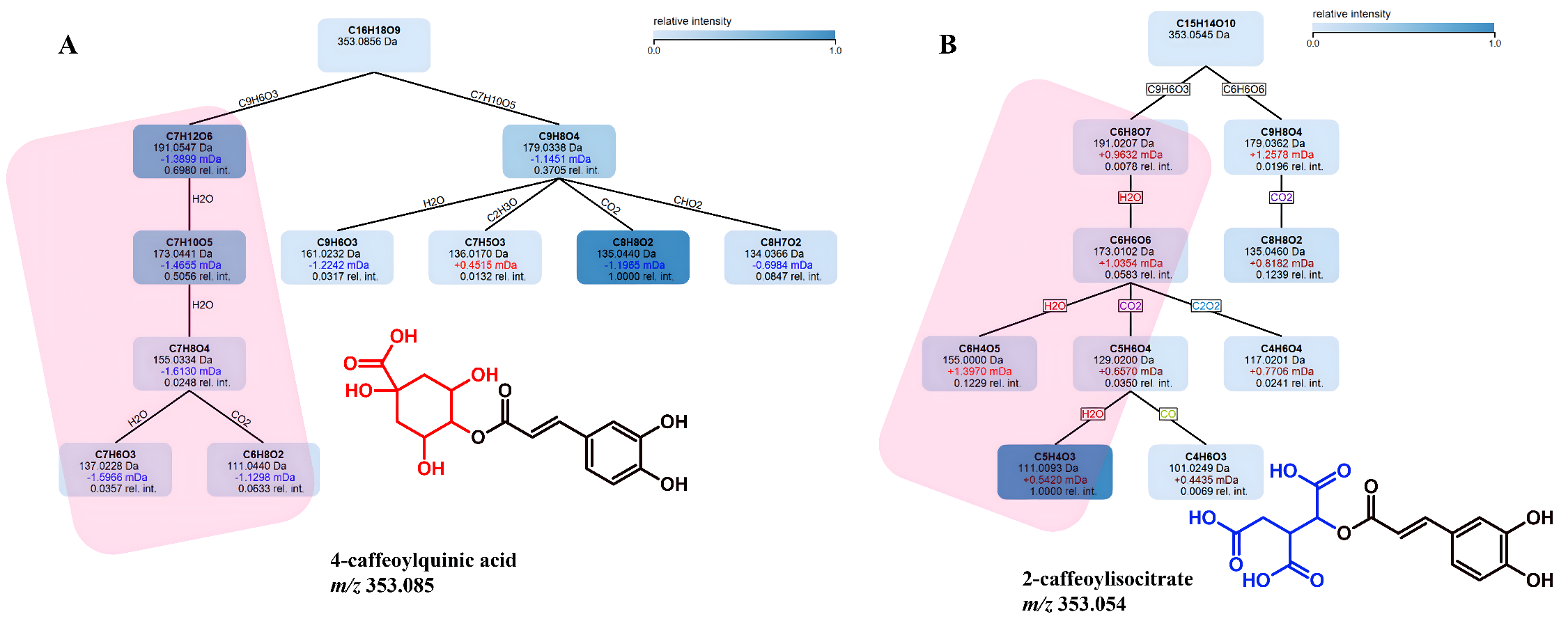
**

**Figure S2** Comparison of the fragmentation patterns of quinic acid in 4-caffeoylquinic acid (A) and isocitric acid in 2-caffeoylisocitrate (B).
